# Incidence trends of ductal carcinoma in situ in New Zealand women between 1999 and 2022

**DOI:** 10.1007/s10549-024-07582-6

**Published:** 2025-01-25

**Authors:** Qian Chen, Mark Elwood, Ian Campbell, Alana Cavadino, Phyu Sin Aye, Sandar Tin Tin

**Affiliations:** 1 Department of Epidemiology and Biostatistics, Faculty of Medical and Health Sciences, University of Auckland, Auckland, New Zealand; 2 Department of Surgery, Faculty of Medical and Health Sciences, University of Auckland, Auckland, New Zealand; 3 Department of Pharmacology, Faculty of Medical and Health Sciences, University of Auckland, Auckland, New Zealand; 4 Cancer Epidemiology Unit, Oxford Population Health, University of Oxford, Oxford, UK

**Keywords:** Ductal carcinoma in situ, Incidence, Trends, Breast screening, Ethnicity, New Zealand

## Abstract

**Background:**

In New Zealand, BreastScreen Aotearoa (BSA), a biennial national breast screening programme, was implemented in 1998. This study examines the incidence trends of ductal carcinoma in situ (DCIS) in New Zealand women from 1999 to 2022.

**Methods:**

All women with a primary diagnosis of DCIS over the 24-year study period were identified from the New Zealand Cancer Registry and BSA records. Age-standardised incidence rates (ASIR), detection rates (ASDR) and average annual percent changes were calculated.

**Results:**

The annual ASIR was 13.5 per 100,000 New Zealand women, and increased by 0.91% (95% confidence interval (CI): 0.26%, 1.66%) annually. Among women aged 45–69 years during 2006–2022, the annual ASIR was 36.3 for programme-detected DCIS, increasing 1.29% (95%CI: 0.13%, 2.73%) per year, and 14.2 for non-programme-detected DCIS, with no significant changes over the study period. The programme-detected ASIRs were highest for Pacific (38.6), Asian (38.2), and Māori (38.0) women. The programme ASDR was 0.55 per 1000 women screened, with no significant changes over time, and was highest for Asian (0.69), and Māori and Pacific (both at 0.65) women.

**Conclusion:**

DCIS incidence increased in New Zealand women from 1999 to 2022, driven by an increase in screening participation, and varied by ethnicity.

**Supplementary Information:**

The online version contains supplementary material available at 10.1007/s10549-024-07582-6.

## Introduction

Ductal carcinoma in situ (DCIS) is a non-invasive precursor lesion to invasive cancer, characterised by abnormal epithelial growth within the breast ducts [1]. Following the introduction of population-based mammographic screening, the incidence of DCIS has risen; for example, the disease now accounts for about 25% of all breast cancer cases (invasive and in situ) identified through screening in the United States [2]. There are also wide variations in incidence trends by age [3], ethnicity [3], and grade [4].

In Aotearoa New Zealand, the biennial national breast screening programme, BreastScreen Aotearoa (BSA), was introduced in 1998 initially for women aged 50 to 64 and extended to include those aged 45 to 69 in 2004 [5]. The number of screened women increased by 40% in the initial two-year period after the age extension, compared to the preceding two years [6]. In a previous report, DCIS accounted for 15% of female breast cancer cases diagnosed in four health regions between 2003 and 2019 [7]. About 80% of all DCIS cases (90% in women aged 45 to 69 years) were detected by mammographic screening, through BSA or private clinics [7]. A more recent report, focusing on DCIS cases in the 45–69 age group diagnosed throughout the country in 2020, found that approximately 70% of DCIS cases were detected through the BSA programme and these cases tended to have smaller median tumour size compared to DCIS detected through non-programme methods [8].

New Zealand is amongst the countries with the highest incidence rate of breast cancer, particularly in indigenous Māori women [9, 10]. The incidence in Pacific women also seems to have increased [10]. However, since the implementation of BSA in 1998, little is known about the DCIS incidence trends in this country. We therefore report the incidence trends of DCIS in New Zealand women overall and by age, ethnicity and detection method over the 24-year period (1999–2022), using data from the New Zealand Cancer Registry (NZCR) and BSA.

## Materials and method

### Data sources

Data from NZCR were linked to BSA records using the National Health Index (NHI) number, a unique identifier assigned to every person who uses health and disability support services in New Zealand. The NZCR is a population-based cancer registry that records all new cancer diagnoses (invasive and in-situ cancer, but excluding non-melanoma skin cancers) in New Zealand [11]. BSA records screening episodes, lesions detected, treatment received and outcomes for all women who participated in the screening programme [12]. Ethics approval for this study was obtained from the Auckland Health Research Ethics Committee (Ref. AH26746).

All women with a primary diagnosis of DCIS between 1 January 1999 and 31 December 2022 were identified from the NZCR, using the International Classification of Diseases 10th revision (ICD-10) diagnosis code “Intraductal carcinoma in situ of breast (D051)”.

Information on age at diagnosis, ethnicity, domicile code, grade, and laterality was extracted. Age at diagnosis was categorised into 5-year age groups for age-standardisation and three age groups: under 45 years, 45–69 years, and 70 years and over to estimate the age-related patterns and trends. Ethnicity was grouped into Māori, Pacific, Asian, European, and others or unknown. The “others or unknown” ethnic group primarily included those who identified themselves as Middle Eastern/Latin American/African (MELAA), other ethnicities, and those who did not state their ethnicity [13]. Patients with more than one recorded ethnicity were allocated to a single ethnic group in order of priority: Māori, Pacific, Asian and European [13]. Neighbourhood deprivation was determined based on the domicile code, using the New Zealand deprivation (NZDep) index (ranging from 1–10 to represent areas from the least to the highest deprived); NZDep 2001 was used for patients diagnosed in 1999–2002, NZDep 2006 for those diagnosed in 2004–2009, NZDep 2013 for those diagnosed in 2010–2015 and NZDep 2018 for those diagnosed in 2016–2022 [14]. Deprivation level was grouped into NZDep 1–4, 5–7 and 8–10. The diagnostic periods were grouped into four periods: 1999–2004, 2005–2010, 2011–2016, and 2017–2022.

### Method of detection

Information on the method of detection was obtained through linkage to the BSA data. DCIS cases were then classified into: (1) programme-detected DCIS, which is defined as DCIS diagnosed within two years after a positive screening examination; (2) non-programme-detected DCIS, which is defined as DCIS diagnosed without meeting criteria for being programme-detected, or diagnosed in women who did not participate in the BSA programme (Supplementary Fig. 1).

### Statistical analyses

Descriptive analyses summarised demographic (age, ethnicity, and diagnostic period) and clinicopathological (tumour grade, tumour laterality) characteristics of women with DCIS overall and by detection method.

The incidence rate of DCIS was calculated as the annual number of cases per 100,000 women, using the New Zealand resident population estimates from 1999 to 2022 [15]. Age-standardised incidence rates (ASIR) were directly standardised to the World Health Organization (WHO) standard population [16]. The rates were presented by age, ethnicity (excluded others or unknown group due to small numbers), tumour grade, detection method and programme screening round. For programme-detected DCIS in women aged 45–69 years, the detection rate was calculated as the number of cases per 1000 programme screened women; this analysis was restricted to those diagnosed between 2006 and 2022 because a substantial number of women from the age extension group started screening in 2006 [6]. Age-standardised detection rates (ASDR) were standardised to the WHO standard population[16]. The programme participation rate was estimated by dividing the number of programme-screened women by the eligible population, with data provided by BSA[17]. To compare the ASIR and ASDR across subgroups defined by age, ethnicity and DCIS grade, incidence rate ratios (IRR) were calculated using women aged 45–69 years, European and those with high-grade DCIS as the reference groups.

Joinpoint regression was used to investigate the potential incidence and detection trends [18]. The programme uses permutation analysis to select the best-fitting regression model with the minimum number of Joinpoints on a logarithmic scale to estimate the annual percentage change (APC) with 95% Confidence Interval (CI) [19]. The average annual percent change (AAPC) was computed as a geometrically weighted average of the generated APCs by the Joinpoint trend analysis software. The weights were equivalent to the length of each segment within a specified time interval [20]. The increase or decrease in incidence and detection trends was considered significant when APCs or AAPCs were statistically significant [21].

An age-period cohort analysis was undertaken by arranging the incidence and population data into the four most recent successive five-year periods, from 2003–2007 to 2018–2022, 5 consecutive five-year age groups from 45–49 years to 65–69 years, and 8 five-year birth cohorts from 1938 to 1973. The period and cohort effects were presented as IRRs using 1938 cohort and period 2003–2007 as the reference groups, respectively. Wald χ2 tests were used to determine which parameters significantly impacted the trends.

Data analyses were performed using R software, version 4.3.2 [22], and Joinpoint trend analysis software from the Surveillance Research Program of the National Cancer Institute (NCI) [18], version 5.1.0. The age-period cohort analysis was conducted using NCI’s Age-Period-Cohort web tool [23]. Statistical significance was defined as P value < 0.05.

## Results

### Patient characteristics

From 1999 to 2022, 9455 DCIS cases were identified, of whom, 5286 (55.9%) were programme-detected (Supplementary Table 1). The median age at diagnosis was 57 years (range 23–98), and the cases were predominantly within the screening age group (45–69 years; 80.7%). The majority of cases were European (7052, 74.6%), and were most commonly diagnosed with high grade (4659, 49.3%). There was an equal distribution of cases in terms of breast laterality. Of the programme-detected DCIS cases, 99.3% were recorded within six months after a positive screening result. Among women aged 45–69 years, 68.9% of DCIS cases were detected through programme.

### Incidence rates of DCIS

The ASIR of DCIS over the study period (1999–2022) was 13.5 per 100,000 New Zealand women per year, with the highest ASIR in those aged 45–69 years. Asian women had the highest ASIR, followed by Māori women. ASIR was highest for high grade DCIS (Table 1).

**Table 1:** Age-standardised incidence rates of DCIS in New Zealand women by age and ethnicity, 1999–2022

Characteristics	Number N (%)	Crude rate			ASIR			ASIR IRR (95% CI)	ASIR AAPC % (95% CI)
		1999	2022	1999–2022	1999	2022	1999–2022 (95% CI)		
Total	9455	11.5	21.3	17.5	10.4	14.9	13.5 (13.2, 13.8)	NA	0.91 (0.26, 1.66)*
Age									
< 45	794 (8.4%)	2.5	2.2	2.4	2.0	1.9	2.0 (1.8, 2.1)	0.04 (0.00, 0.11) *	− 0.31 (− 1.21, 0.55)
45–69	7629 (80.7%)	36.1	56.1	49.1	36.8	54.1	48.7 (47.6,49.8)	1 (Reference)	0.99 (0.18,1.92)*
≥ 70	1032 (10.9%)	10.8	24.2	18.6	12.0	25.7	19.9 (18.7, 21.2)	0.41 (0.34, 0.47)*	1.70 (0.36, 3.37)*
Ethnicity^a^									
Māori	952 (10.1%)	6.2	16.8	11.2	9.4	16.8	13.1 (12.2, 13.9)	1.04 (0.98, 1.11)	1.10 (− 0.15, 2.67)
Pacific	375 (4.0%)	4.0	15.0	9.2	6.0	18.4	12.2 (11.0, 13.6)	0.98 (0.87, 1.08)	2.12 (0.05, 5.07)*
Asian	896 (9.5%)	3.2	17.2	13.8	5.1	16.7	13.7 (12.8, 14.6)	1.10 (1.02, 1.17)*	1.56 (0.63, 2.89)*
European	7052 (74.6%)	11.7	20.1	17.5	9.9	12.5	12.5 (12.2, 12.8)	1 (Reference)	0.23 (− 0.71, 1.37)
DCIS grade									
Low	1221 (12.9%)	1.3	2.4	2.3	1.1	1.6	1.7 (1.6, 1.8)	0.28 (0.26, 0.31)*	− 2.43 (− 3.63, − 1.20)*
Intermediate	3227 (34.1%)	3.3	6.9	6.0	3.1	4.7	4.6 (4.5, 4.8)	0.71 (0.69, 0.72)*	0.93 (− 0.35, 2.40)
High	4659 (49.3%)	4.5	11.2	8.6	4.1	8.2	6.7 (6.5, 6.9)	1 (Reference)	2.49 (1.58, 3.52)*
Unknown	348 (3.7%)	2.3	0.8	0.6	2.1	0.5	0.5 (0.4, 0.5)	0.11 (0.06, 0.16)*	− 5.92 (− 8.45, − 4.20)*

*AAPC* average annual percentage change, *ASIR* age-standardised incidence rates, *NA* not applicable, *IRR* incidence rate ratios

*Indicates the IRR, AAPC is statistically significant

^a^180 cases with other or unknown ethnicity were excluded

**Fig. 1:**
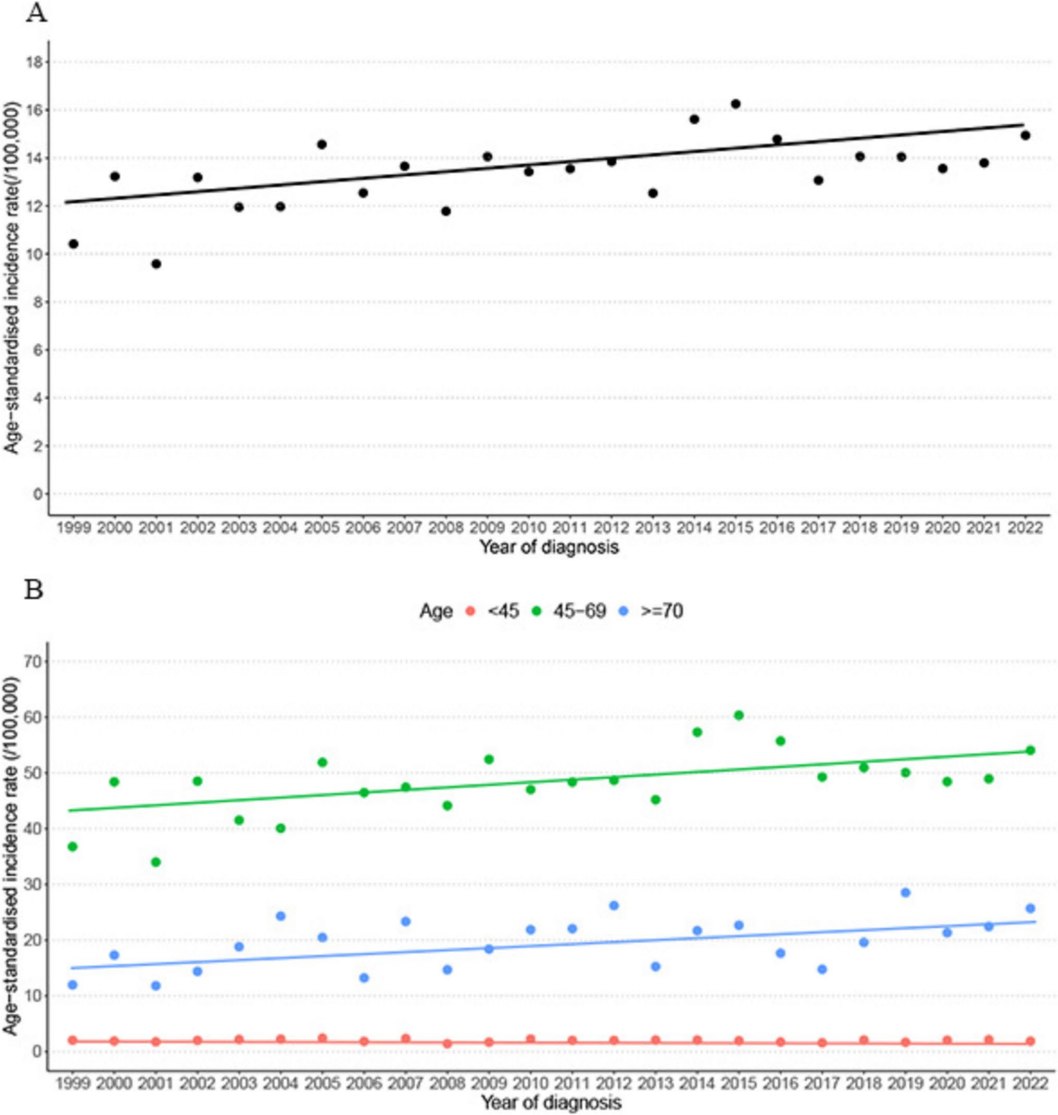
Annual age-standardised incidence rates of DCIS from 1999 to 2022 in all women (**A**), and by age group (**B**), and fitted logarithmic trends

Over the study period, DCIS incidence increased by 0.91% (95% CI: 0.26%, 1.66%) annually, which was driven by the increasing trends in women aged 45–69 and over 70 (Fig. 1). The increase was most pronounced among the Pacific and Asian women. ASIR increased for high grade DCIS but decreased for low and unknown grades (Table 1).

### Temporal trends in DCIS incidence among women aged 45–69 years

The age-specific incidence of programme-detected DCIS in women aged 45–49 and 65–69 increased dramatically after screening age expansion in 2004, while the incidence of non-programme-detected DCIS slightly decreased over time for all screening age groups (Supplementary Fig. 2).

The age-period-cohort analysis of women with DCIS aged 45–69 showed a marginal period effect (P = 0.0856) (Supplementary Fig. 3). Incidence rates by calendar periods rose and peaked in the period from 2013 to 2017 (IRR, 1.20; 95% CI 1.04 to 1.39).

### Incidence rates of DCIS among women aged 45–69 years by detection method

From 2006 to 2022, among women of screening age (45–69 years), 4375 (72%) were detected through the programme. The ASIR was higher for cases detected through programme (36.3 per 100,000 women) compared to those detected through other routes (14.2) (Table 2).

**Table 2:** Age-standardised incidence rates of DCIS among women aged 45–69 years, 2006–2022, by detection method, age, ethnicity and tumour grade

Characteristics	Number	Crude rate			ASIR			ASIR IRR (95% CI)	ASIR AAPC % (95% CI)
		2006	2022	2006–2022	2006	2022	2006–2022 (95% CI)		
Programme-detected									
Total	4375	28.0	40.0	36.7	27.9	38.4	36.3 (35.2, 37.4)	NA	1.29 (0.13, 2.73)*
Ethnicity^a^									
Māori	510	33.5	43.8	37.5	34.2	44.7	38.0 (34.8, 41.5)	1.16 (1.07, 1.26)*	0.71 (− 1.24, 3.01)
Pacific	213	25.3	54.4	37.6	26.4	57.4	38.6 (33.6, 44.2)	1.18 (1.04, 1.32)*	3.14 (− 0.76, 8.44)
Asian	485	16.1	37.8	38.1	15.7	38.2	38.2 (34.9, 41.8)	1.17 (1.07, 1.27)*	6.31 (3.96, 10.70)*
European	3084	27.1	32.3	32.6	26.8	30.9	32.6 (31.5, 33.8)	1 (Reference)	0.17 (− 1.35, 1.79)
DCIS grade^b^									
Low	434	4.4	4.2	3.6	4.4	3.9	3.6 (3.3, 4.0)	0.18 (0.08, 0.29)*	− 1.10 (− 2.57, 0.39)
Intermediate	1509	10.0	12.5	12.7	10.0	11.9	12.6 (12.0, 13.2)	0.60 (0.57, 0.70)*	0.72 (− 1.49, 3.60)
High	2398	12.6	22.4	20.1	12.6	21.7	19.8 (19.0, 20.6)	1 (Reference)	2.01 (0.62, 3.87)*
Non-programme-detected									
Total	1692	18.6	16.1	14.2	18.6	15.7	14.2 (13.5, 14.9)	NA	− 1.09 (− 3.01, 1.01)
Ethnicity^c^									
Māori	172	10.6	15.9	12.6	9.8	16.1	12.9 (11.0, 15.0)	0.96 (0.80, 1.12)	− 1.01 (− 5.34, 3.98)
Pacific	61	12.7	11.8	10.8	12.6	13.1	11.0 (8.4, 14.2)	0.82 (0.56, 1.08)	− 0.62 (− 5.58, 5.16)
Asian	182	30.0	17.4	14.3	27.1	18.0	14.4 (12.4, 16.7)	1.08 (0.92, 1.23)	− 2.31 (− 6.72, 3.01)
European	1249	17.8	13.8	13.4	17.9	13.6	13.4 (12.7, 14.2)	1 (Reference)	− 1.54 (− 3.74, 0.87)
DCIS grade^d^									
Low	250	4.3	2.3	2.1	4.3	2.3	2.1 (1.8, 2.3)	0.30 (0.16, 0.44)*	− 4.74 (− 7.06, − 1.74)*
Intermediate	554	6.6	4.6	4.7	6.7	4.5	4.7 (4.3, 5.1)	0.68 (0.57, 0.79)*	− 1.11 (− 4.69, 2.93)
High	829	6.5	8.5	7.0	6.5	8.3	6.9 (6.5, 7.4)	1 (Reference)	0.18 (− 1.78, 2.27)

*AAPC* average annual percentage change, *ASIR* age-standardised incidence rates, *NA* not applicable, *IRR* incidence rate ratios

*Indicates the IRR, AAPC is statistically significant

^a^In programme-detected, 83 cases with other or unknown ethnicity were excluded

^b^In programme-detected, 34 cases with unknown tumour grade were excluded

^c^In non-programme-detected, 18 cases with other or unknown ethnicity were excluded

^d^In non-programme-detected, 59 cases with unknown tumour grade were excluded

For programme-detected DCIS, compared to European, other ethnicities had significantly higher ASIR (Pacific: IRR, 1.18 (95% CI 1.04, 1.32); Asian: IRR, 1.17 (95% CI 1.07,1.27); Māori: IRR, 1.16 (95% CI 1.07,1.26)) (Table 2).

**Fig. 2:**
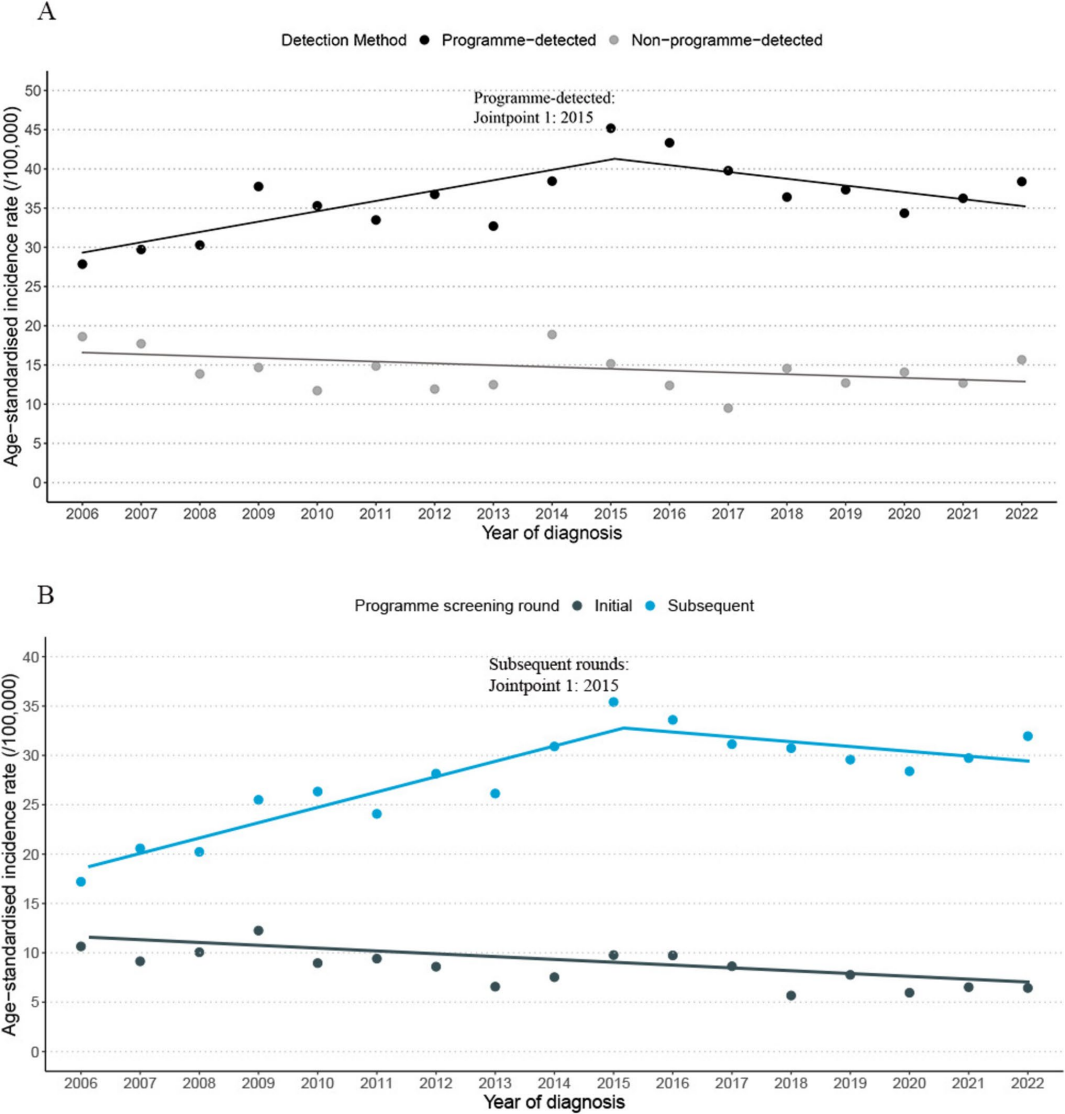
Age-standardised incidence rate of DCIS among women aged 45–69 years from 2006 to 2022, by detection method (**A**), and programme screening round (**B**)

Programme-detected DCIS incidence increased over the study period by 1.29% (95% CI 0.13%, 2.73%) (Table 2). Incidence increased from 2006 to 2015 (APC, 4.04%; 95% CI 2.23%, 10.62%) and showed a non-significant decrease afterwards (APC, − 2.13%; 95% CI − 8.05%, 0.20%) (Fig. 2A). The incidence of DCIS detected in the subsequent screening rounds increased by 2.81% (1.63%, 4.31%), while the incidence in the initial round significantly decreased (AAPC: − 3.12%; − 5.06%, − 1.21%) (Fig. 2B). Over the whole period, this increase was most pronounced for Asian women (AAPC, 6.31%; 95% CI 3.96%, 10.70%) and for high-grade DCIS (AAPC, 2.01%; 95% CI 0.62%, 3.87%).

For non-programme-detected cases, the incidence remained stable over the study period and did not differ by ethnicity (Table 2).

### Programme detection rates for DCIS among women aged 45–69 years

Between 2006 and 2022, the ASDR for women aged 45–69 years was 0.55 per 1000 women screened. Asian women had the highest ASDR (0.69), followed by Māori and Pacific (both at 0.65), all significantly higher than that of European women (Asian: IRR, 1.36 (95% CI 1.27, 1.46); Māori: IRR, 1.29 (95% CI 1.20,1.39); Pacific: IRR, 1.28 (95% CI 1.14, 1.42)) (Table 3).

**Table 3:** Age-standardised detection rates for DCIS among women aged 45–69 years, 2006–2022, overall, by ethnicity, by tumour grade

Characteristics	Crude rate			ASDR			ASDR IRR (95% CI)	ASDR AAPC % (95% CI)
	2006	2022	2006–2022	2006	2022	2006–2022 (95% CI)		
Total	0.57	0.60	0.56	0.63	0.58	0.55 (0.54, 0.57)	NA	− 0.11 (− 1.08, 0.98)
Ethnicity^a^								
Māori	0.98	0.72	0.65	1.06	0.71	0.65 (0.60, 0.71)	1.29 (1.20, 1.39)*	− 3.50 (− 5.49, − 0.74)*
Pacific	0.91	0.91	0.64	0.75	0.89	0.65 (0.56, 0.74)	1.28 (1.14, 1.42)*	0.34 (− 2.97, 4.79)
Asian	0.58	0.67	0.69	0.66	0.65	0.69 (0.63, 0.76)	1.36 (1.27, 1.46)*	0.98 (− 0.92, 6.22)
European	0.53	0.53	0.51	0.58	0.50	0.51 (0.49, 0.52)	1 (Reference)	− 0.22 (− 1.51, 1.21)
DCIS grade^b^								
Low	0.09	0.06	0.06	0.10	0.06	0.06 (0.05, 0.06)	0.18 (0.08, 0.29)*	− 2.67 (− 4.75, − 0.38)*
Intermediate	0.21	0.19	0.19	0.23	0.18	0.19 (0.18, 0.20)	0.64 (0.57, 0.70)*	− 0.76 (− 2.30, 0.94)
High	0.26	0.34	0.31	0.27	0.33	0.30 (0.29, 0.31)	1 (Reference)	0.86 (− 0.12, 1.98)

*AAPC* average annual percentage change, *ASDR* age-standardised detection rates, *NA* not applicable, *IRR* incidence rate ratios

*Indicates the IRR, AAPC is statistically significant

^a^83 cases with other or unknown ethnicity were excluded. The programme detection rate by ethnicity were based on the data from Māori, Pacific, Asian, and Other (As European account approximately 99% of the ‘Other’ ethnicity, we used the screened and eligible population of ‘Other’ from BSA to represent European)

^b^34 cases with unknown tumour grade were excluded

*Note* The screening eligible population estimate data source: New Zealand Census Statistics (incidence rate), BSA data (detection rate and programme participation rate)

The programme detection rate remained stable over the study period, but a significant decrease was observed in Māori women (AAPC, − 3.50%; 95% CI − 5.49%, − 0.74%), and low-grade DCIS (AAPC, − 2.67%; 95% CI − 4.75%, − 0.38%) (Table 3).

**Fig. 3:**
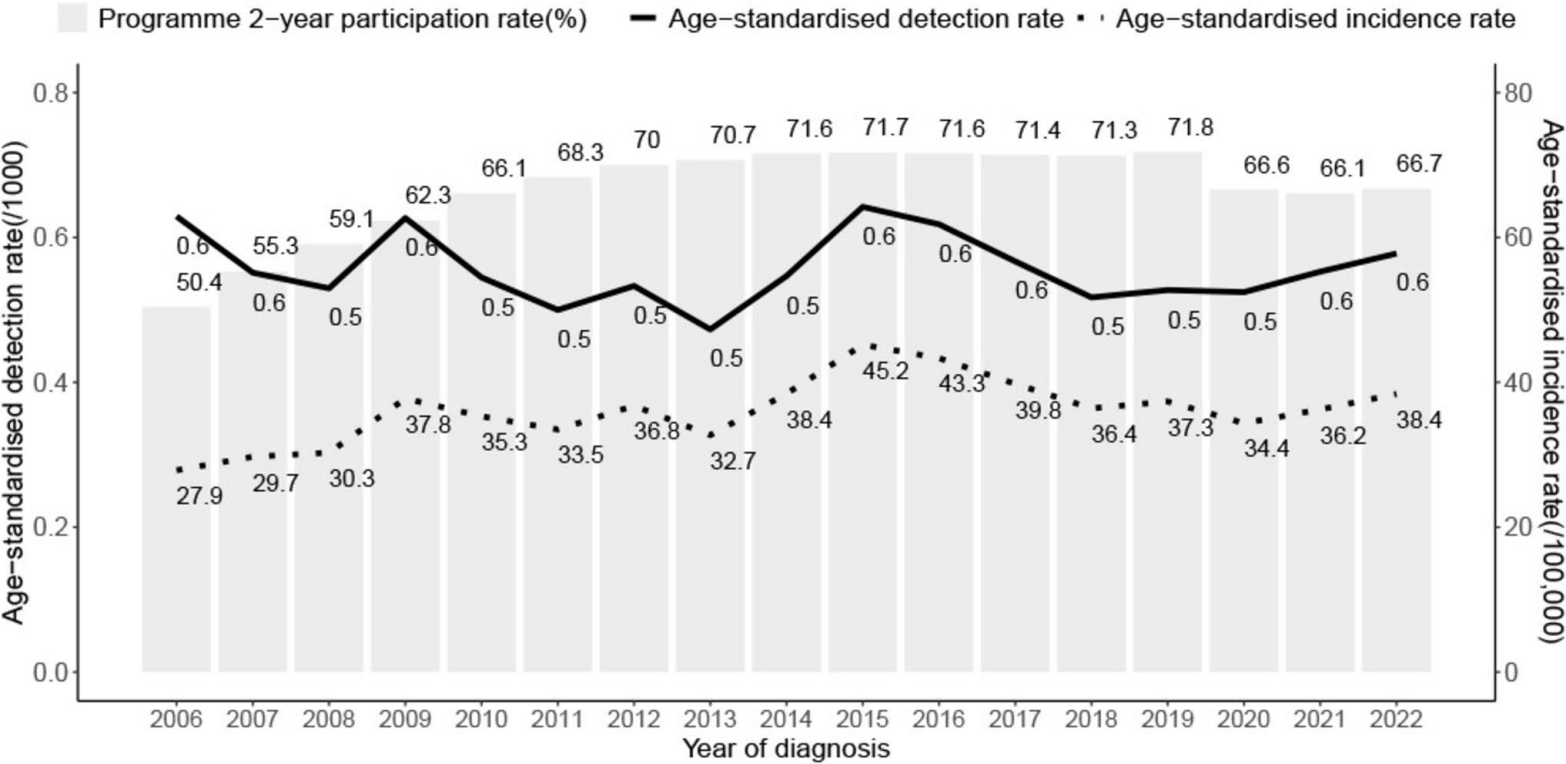
Age-standardized detection rate, programme-detected DCIS age-standardized incidence rate and programme 2-year participation rate, among women aged 45–69 years, 2006–2022

As the programme participation rates improved over time, the programme-detected DCIS incidence increased, despite a relatively stable detection rate for all women aged 45–69 years (Fig. 3). However, these trends varied across ethnic groups, with a larger disparity between programme detection and incidence rates observed in Asian and Māori women (Supplementary Fig. 4).

Age-specific detection rates were higher for the 60–64 and 65–69 year age groups, at 0.61 and 0.68 per 1000 women screened, respectively (Supplementary Fig. 5). Moreover, the high-grade DCIS detection rate increased with age.

## Discussion

Our analysis showed that the annual ASIR of DCIS was 13.5 per 100,000 New Zealand women; the incidence increased from 1999 to 2022. Among women aged 45–69 years, programme-detected DCIS incidence increased over time, while non-programme-detected DCIS incidence and programme detection rates remained stable. Asian, Pacific and Māori women had the highest programme-detected DCIS incidence and detection rates.

The annual ASIR of DCIS was highest (48.7) among those aged 45–69 years between 1999 and 2022. These findings align with studies from other countries, which reported incidence rates ranging from 10 to 30 per 100,000 women overall [3, 24–31] and from 40 to 70 in the screening age group [3, 24–26]. In our analysis, the DCIS incidence increased by an average of 0.91% annually, driven by a 0.99% increase in women aged 45–69 and a 1.70% increase in women over 70 years per year. In an earlier study from the United States, DCIS incidence rates continued to increase for women aged 70 to 79 years from 1992 to 2011, while the rates for those aged 50 to 69 years plateaued after 1999 [32], indicating the need to consider the potential impact of diagnosing more DCIS by extending screening to include older women [33].

Among women aged 45–69 years, 72.0% of DCIS cases were detected through the screening programme during 2006–2022. Likewise, population-based studies from various countries have shown that screen-detected DCIS accounts for 76–78% of all DCIS cases among screening-eligible age groups [26, 34]. In our study, programme-detected DCIS incidence increased significantly from 2006 to 2015, followed by a non-significant decrease, similar to previous research demonstrating a dramatic increase in DCIS incidence after the introduction of mammographic screening, followed by stabilization within 10–20 years [3, 29, 31, 35]. Notably, the mammogram participation rates of screening eligible women became stable after 1998 in the United States, highlighting the impact of breast screening on the DCIS incidence [36]. The presence of marginal period effects further suggests that programme screening and advancements in detection technology may play a role in increasing DCIS incidence. Additionally, the increased programme-detected DCIS incidence was driven by the subsequent screening rounds, likely reflecting the increasing number of women receiving screening in the subsequent rounds.

We compared programme detection rate and programme-detected incidence rate to help determine whether changes in DCIS incidence were driven by screen-detection or screening participation. The screening programme has set a ‘target’ proportion of DCIS among BSA programme-detected breast cancer cases as 10–25% for those aged 50–69 years, with a reported proportion of 24% from 2011 to 2016 [37]. The increasing programme-detected incidence rate, despite stable DCIS programme detection rates observed in our study, shows that DCIS incidence is primarily influenced by the programme participation rates. In New Zealand, the breast screening programme aims to cover 70% of eligible women [38]. The national participation rate has increased over time, from 50.4% in 2006 to 71.8% in 2015, then remained stable until the COVID-19 pandemic, and declined to 65.2% in 2022 [17].

Significant ethnic disparities in programme-detected DCIS were found in our study, with Asian, Pacific and Māori women exhibiting higher incidence rates compared to European. Among these ethnic groups, Asian women showed the most substantial increase in DCIS incidence. For non-programme detected DCIS, although ethnic differences were not significant, Asian had the highest incidence. Moreover, Māori and Asian women had lower participation rates compared to other ethnicities (primarily European) [17]. A similar ethnic disparity pattern was observed in the United States, where Asian-Pacific Islander, African American and Hispanic women, despite having lower screening rates, exhibited higher DCIS incidence rates compared to European Americans [39]. These findings suggest that although screening participation substantially influences the DCIS incidence, it does not fully explain the higher incidence in certain ethnic groups.

The average detection rate of carcinoma in situ (ductal and lobular) in the 50–69 years groups was 1.01 per 1000 women screened in Europe [40]. For this age group, DCIS detection rates range from 0.45 in Finland to 1.55 in Denmark per 1000 women screened, with a higher detection rate in older age groups (0.68 for those aged 50–59 years, 0.83 for those aged 60–69 years) [41]. The increased DCIS detection rate with age could be due to the increase in high-grade DCIS [42]. Asian and African American women were reported to have the higher DCIS detection rates than European in the United States, while Hispanic women had lower rates [43]. In New Zealand, the DCIS detection rate was 0.55 per 1000 screened women between 2006 and 2022. This rate is slightly lower than other countries, but the rates follow a similar pattern, increasing with age and tumour grade. We also found Asian women had the highest DCIS detection rate, followed by Māori and Pacific women. While previous research has discussed reproductive behaviours and biological features related to ethnic disparities in invasive breast cancer in New Zealand [44], reasons for these differences in DCIS needs further exploration.

We observed that nearly half of DCIS were high-grade, with a higher proportion among programme-detected cases compared to non-programme-detected cases. The proportion of unknown-grade DCIS cases decreased over time, indicating improvement in pathological assessment. In programme-detected cases, we observed a substantial increase in high-grade DCIS incidence, while low-grade DCIS declined. Conversely, non-programme-detected cases showed a significant decrease in low-grade DCIS incidence. Our findings align with other studies which reported that 40–60% of DCIS cases at diagnosis were high-grade, followed by intermediate grade at approximately 40% [3, 4, 7, 45–50]. Additionally, screen-detected cases tended to have a slightly higher proportion of high-grade DCIS than non-screen-detected cases [4, 51, 52]. The adoption of digital mammography, which enhances sensitivity for calcification compared to film-screen mammography, has been associated with increased detection of high-grade DCIS, with little impact on low-grade DCIS[53, 54]. In New Zealand, digital mammography was introduced in 2006 with the nationwide implementation completed in 2013[55]. Moreover, the diagnosis of low-grade DCIS requires more caution from pathologists compared to high-grade DCIS [56]. The reduction in low-grade DCIS is somewhat reassuring, given the debate over the natural history of low-grade DCIS and uncertainties regarding the degree to which diagnosis of low-grade DCIS contributes to over-diagnosis, and over-treatment in screening programmes.

To the best of our knowledge, our study is the first to provide a comprehensive, national-level evaluation of DCIS incidence trends across the entire population of Aotearoa New Zealand over 24 years. We were able to analyse both population-based incidence rates and programme-based detection rates. However, due to limited data availability, we were unable to investigate the possible effects of digital versus film mammography and private breast screening on the DCIS incidence. The detection rate by screening rounds could not be assessed as we do not have data on participation by rounds. Moreover, we do not have information on individual-level programme participation and potential risk factors, which limits our ability to fully explain the differences observed between ethnic groups.

In conclusion, this population-based study reveals a significant increase in DCIS incidence rates in New Zealand, primarily driven by the increase in screening. The detection rate of DCIS in screened women and the incidence of non-programme-detected cases remained stable over time. For programme-detected DCIS, Asian, Pacific and Māori women had a higher DCIS incidence compared to the majority European group, and Asian women experienced the greatest increase. Our findings underscore the need for further efforts to understand ethnic-specific risk factors for DCIS and to improve accessibility to the screening programme for all women, including appropriate pathways for Māori, Pacific and Asian women.

## Supplementary Information

Below is the link to the electronic supplementary material.

Supplementary file1 (DOCX 161 KB)

## Data Availability

The data for this study were provided by the New Zealand Ministry of Health, and may be available to other researchers who meet data access requirements. For more information on eligibility and data access, please contact the Ministry of Health at data_enquiries@moh.govt.nz.
