# Supplementary material for: Incidence trends of ductal carcinoma in situ in New Zealand women between 1999 and 2022
Source: Breast Cancer Res Treat. 2025 Jan 25;210(2):439–49. doi: 10.1007/s10549-024-07582-6 (PMC11930874; doi:10.1007/s10549-024-07582-6)
Supplement: Supplementary file 1 — Supplementary file1 (DOCX 161 KB) [file 10549_2024_7582_MOESM1_ESM.docx]

Supplementary materials


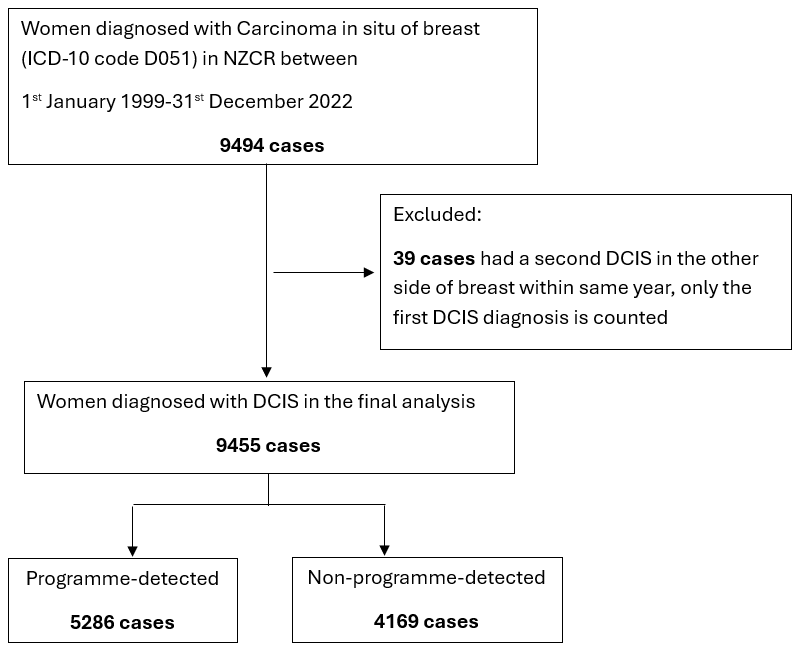


Abbreviations: DCIS: ductal carcinoma in situ; NZCR: New Zealand Cancer Registry

Supplementary Figure 1. Flow diagram of the identification of eligible cases

Supplementary Table 1 Characteristics of women diagnosed with DCIS from 1999 to 2022, by detection method.

|  | **Overall** | **Programme-detected** | **Non-programme-detected** | **P ^b^** |
| --- | --- | --- | --- | --- |
| N (%) | 9455 | 5286 | 4169 |  |
| Age (median, range) | 57 (23, 98) | 57 (45, 71) | 56 (23, 98) | 0.5743 |
| Age group |  |  |  | <0.0001 |
| <45 years | 794 (8.4%) | 0 (0.0%) | 794 (19.0%) |  |
| 45-69 years | 7,629(80.7%) | 5,256 (99.4%) | 2,373 (56.9%) |  |
| ≥70 years | 1,032(10.9%) | 30 (0.6%) | 1,002 (24.0%) |  |
| Ethnicity |  |  |  | <0.0001 |
| Māori | 952(10.1%) | 605 (11.4%) | 347 (8.3%) |  |
| Pacific | 375 (4.0%) | 233 (4.4%) | 142 (3.4%) |  |
| Asian | 896 (9.5%) | 536 (10.1%) | 360 (8.6%) |  |
| European | 7052 (74.6%) | 3,823 (72.3%) | 3,229 (77.5%) |  |
| Other or Unknown | 180 (1.9%) | 89 (1.7%) | 91 (2.2%) |  |
| NZ Deprivation ^a^ |  |  |  | <0.0001 |
| 1-4 | 4,119 (43.6%) | 2,189 (41.4%) | 1,930 (46.4%) |  |
| 5-7 | 2,858 (30.3%) | 1,626 (30.8%) | 1,232 (29.6%) |  |
| 8-10 | 2,465 (26.1%) | 1,467 (27.8%) | 998 (24.0%) |  |
| Diagnosis year |  |  |  | <0.0001 |
| 1999-2004 | 1,642 (17.4%) | 698 (13.2%) | 944 (22.6%) |  |
| 2005-2010 | 2,198 (23.2%) | 1,199 (22.7%) | 999 (24.0%) |  |
| 2011-2016 | 2,699 (28.5%) | 1,634 (30.9%) | 1,065 (25.5%) |  |
| 2017-2022 | 2,916 (30.8%) | 1,755 (33.2%) | 1,161 (27.8%) |  |
| Tumour grade |  |  |  | <0.0001 |
| Low | 1,221 (12.9%) | 604 (11.4%) | 617 (14.8%) |  |
| Intermediate | 3,227(34.1%) | 1,832 (34.7%) | 1,395 (33.5%) |  |
| High | 4,659 (49.3%) | 2,761 (52.2%) | 1,898 (45.5%) |  |
| Unknown | 348 (3.7%) | 89 (1.7%) | 259 (6.2%) |  |
| Laterality |  |  |  | 0.0408 |
| Left | 4,898 (51.8%) | 2,754 (52.1%) | 2,144 (51.4%) |  |
| Right | 4,497 (47.6%) | 2,508 (47.4%) | 1,989 (47.7%) |  |
| Unknown | 60 (0.6%) | 24 (0.5%) | 36 (0.9%) |  |

NZ Deprivation: New Zealand deprivation index (1 to 10 to represent areas from the least to the most deprived).

a. 13 cases with no deprivation index recorded were excluded.

b. P values are from Wilcoxon rank sum test for age, from Chi squared tests for other categorical variables.


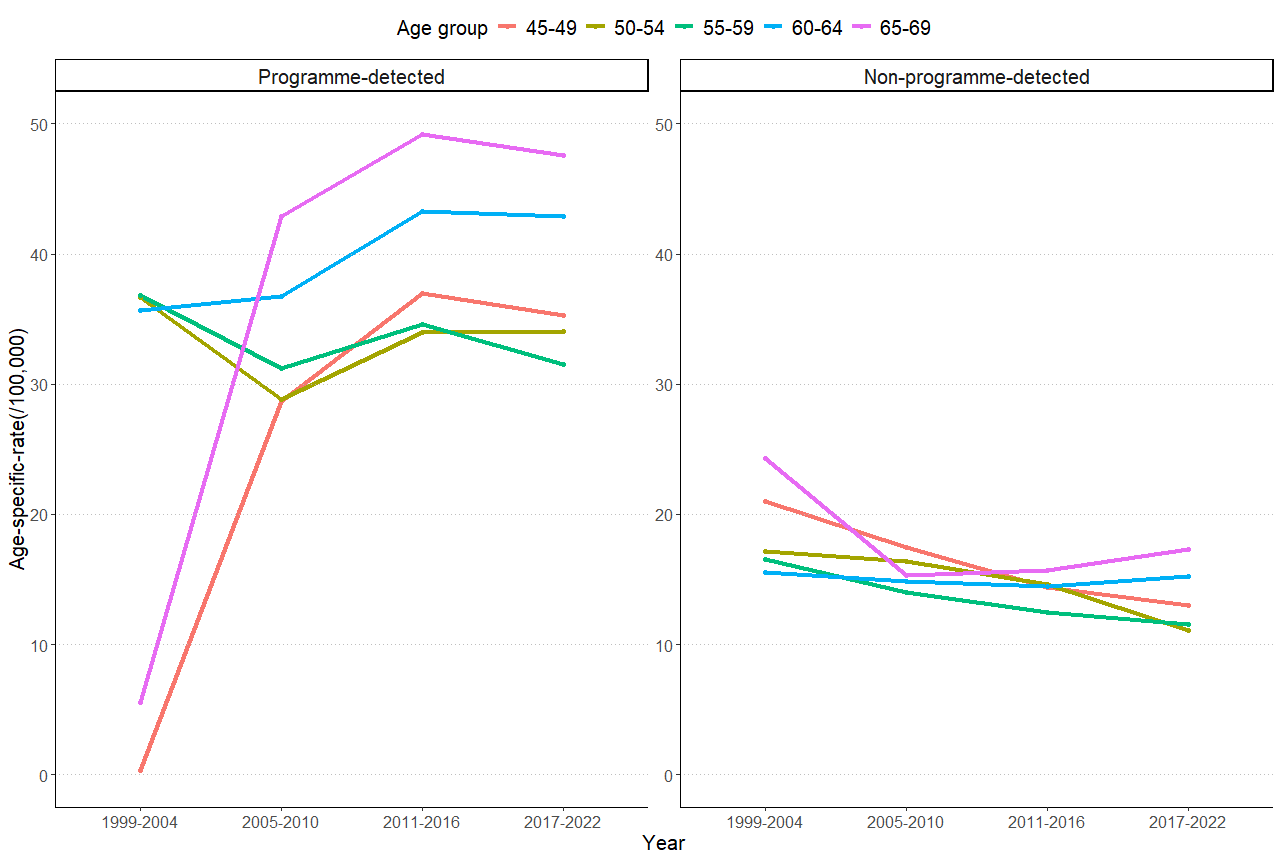


Supplementary Figure 2. Age-specific incidence rates of DCIS among women aged 45-69 years, 1999-2022, by detection method.


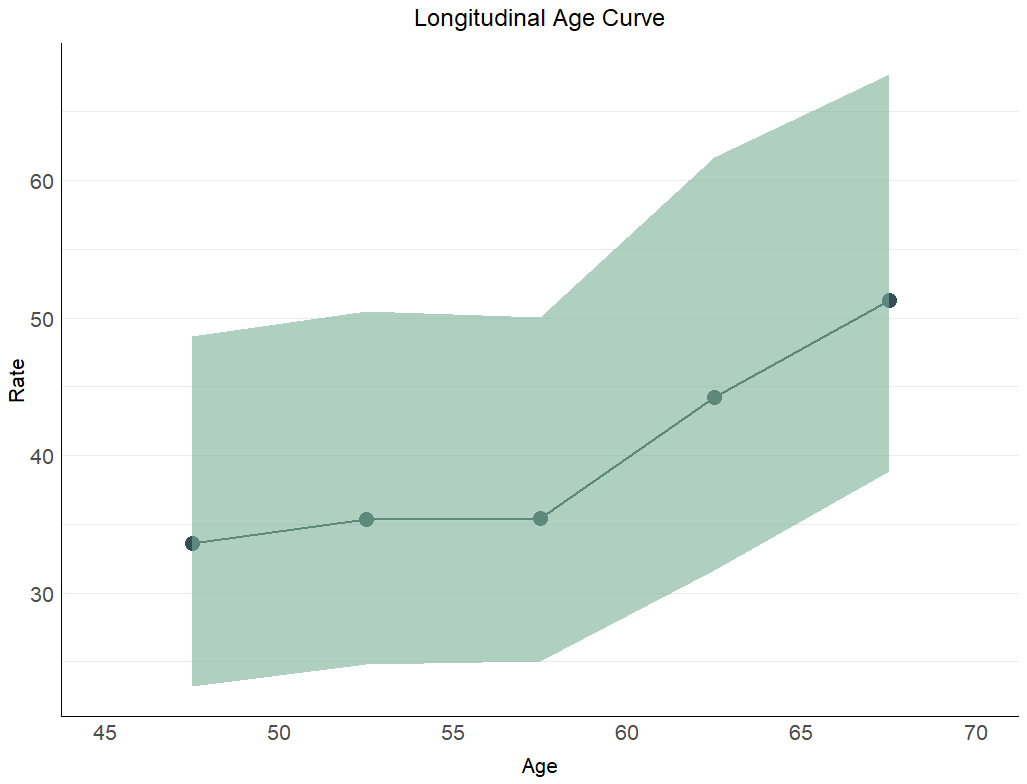

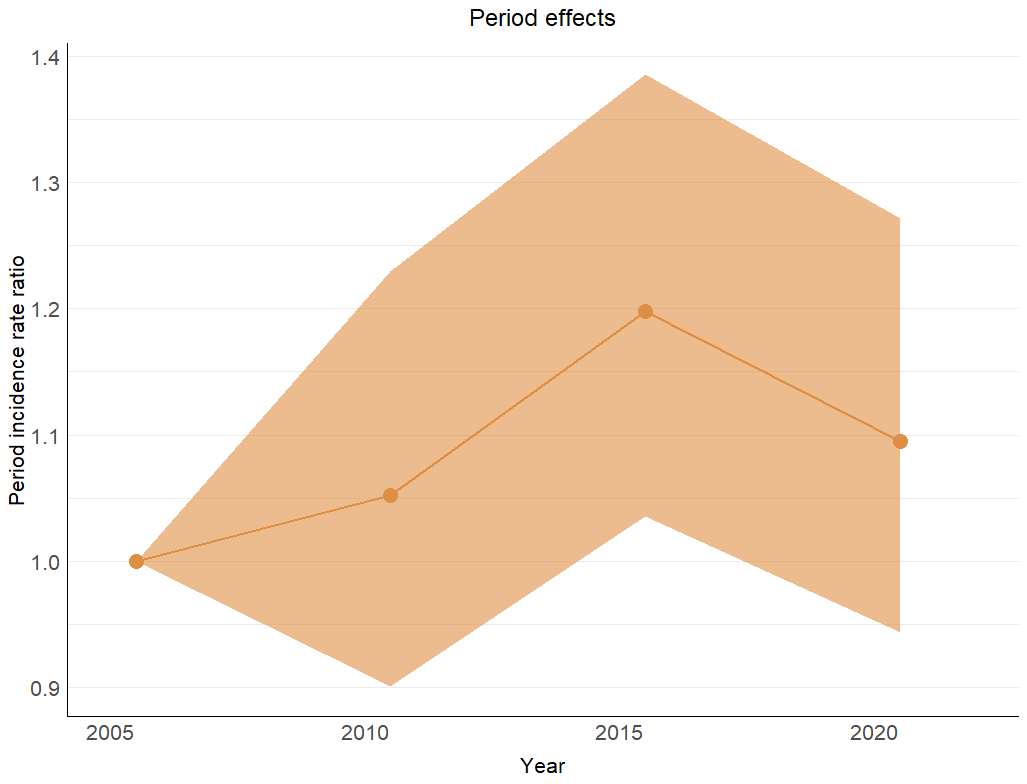

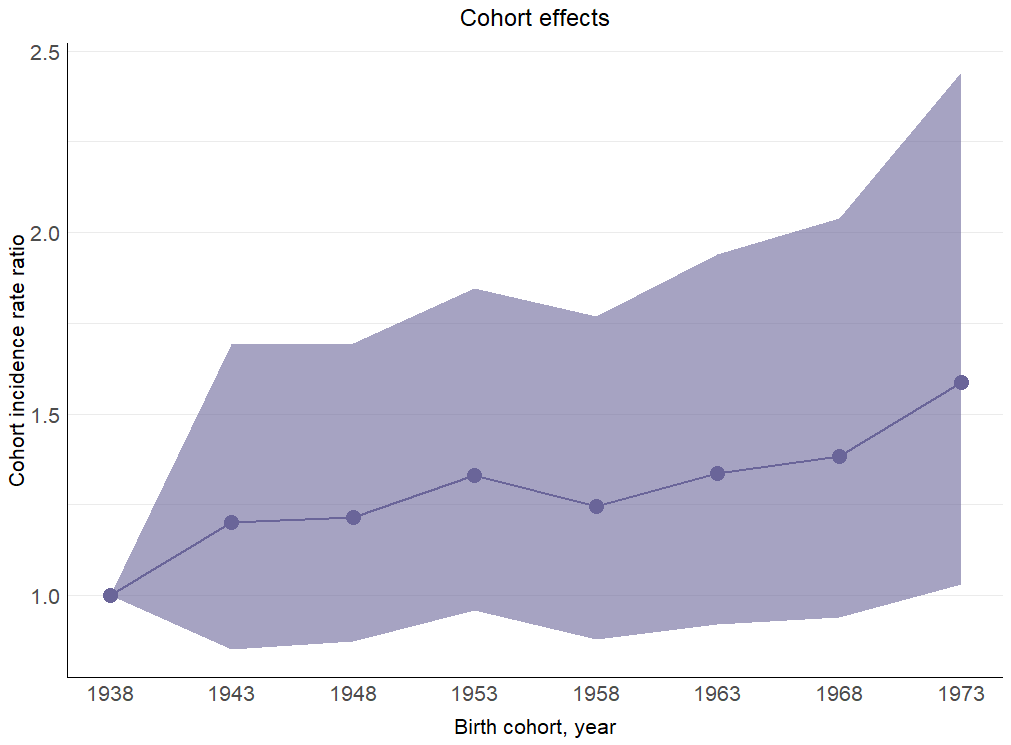


Note: Shaded areas indicate 95% CIs. In the Period rate ratio (RR) figure, the years represent the following time periods: 2005 (2003-2007), 2010 (2008-2012), 2015 (2013-2017), and 2020 (2018-2022).

Supplementary Figure 3. Age-period-cohort analysis on DCIS incidence among women aged 45-69 years, from 2003 to 2022.


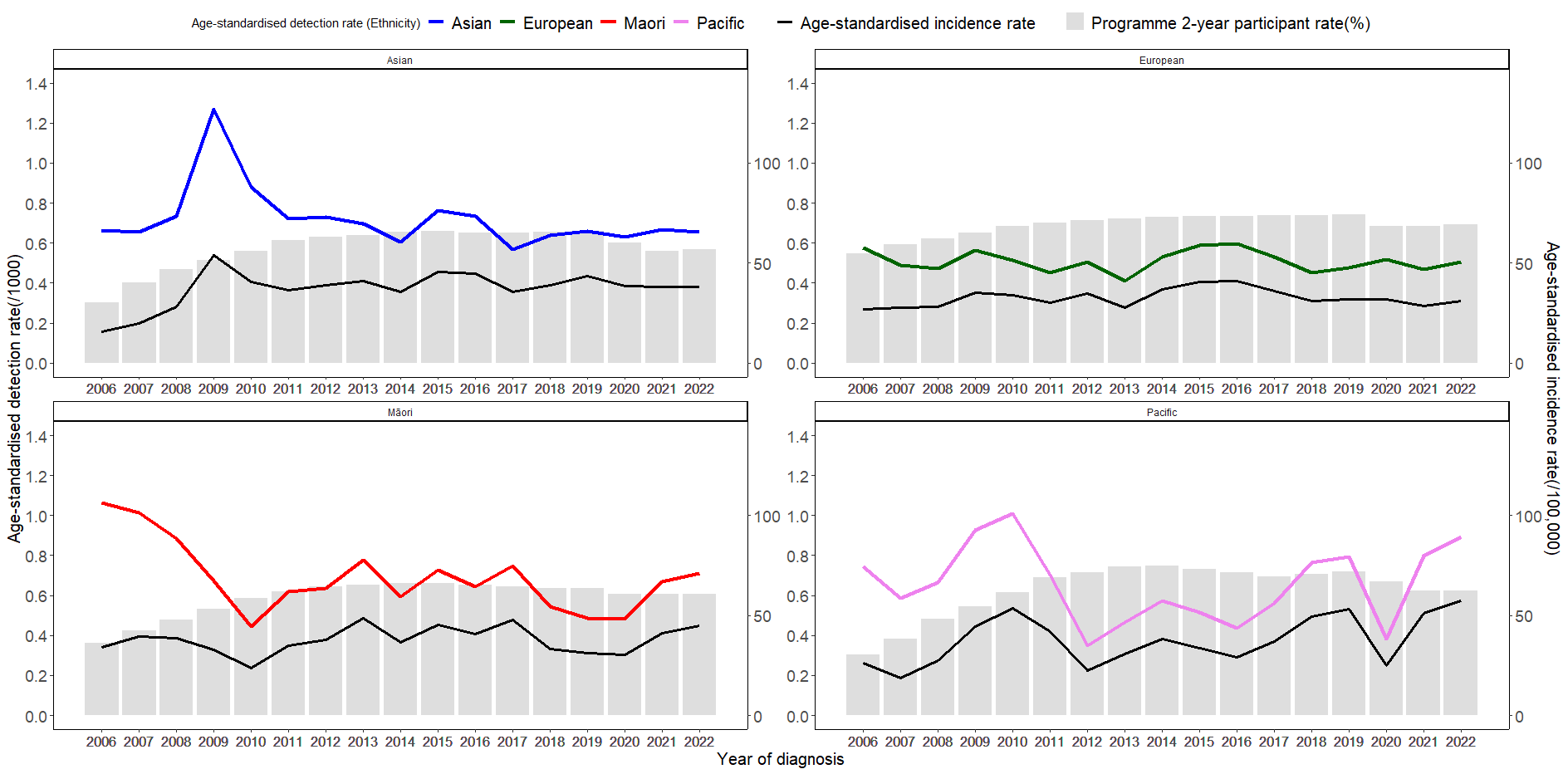


Note: The programme detection rate and coverage by ethnicity were based on the data from Māori, Pacific, Asian, and Other (As European account approximately 99% of the ‘Other’ ethnicity, we used the screened and eligible population of ‘Other’ from BSA to represent European). The programme 2-year participation (%) was represented using the same ASIR y-axis.

Supplementary Figure 4. Age-standardized detection rate (colour line), programme-detected DCIS age-standardized incidence rate (black line) and programme 2-year coverage rate (bar), among women aged 45-69 years, 2006-2022, by ethnicity.


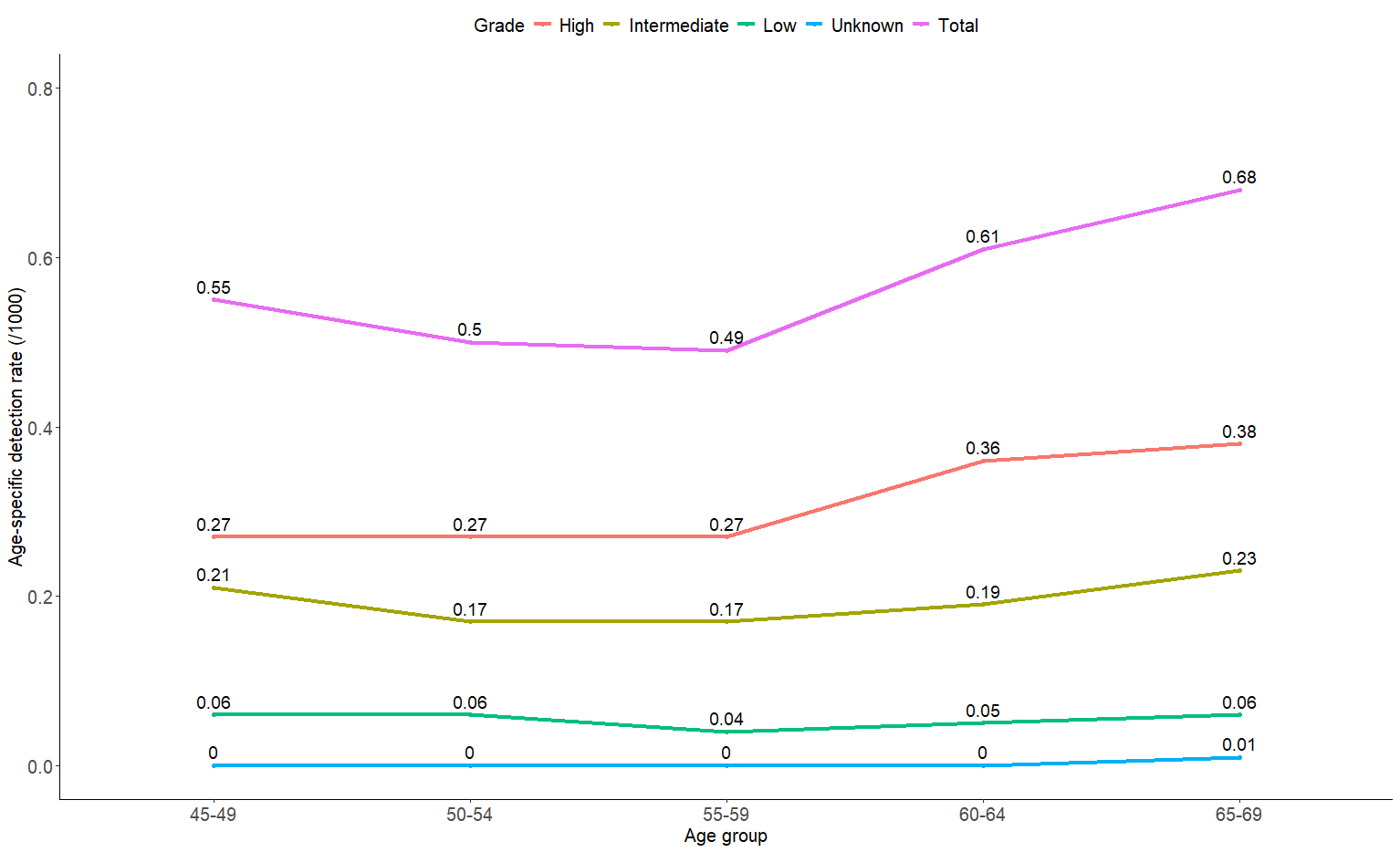


Supplementary Figure 5. Age-specific detection rate of DCIS per 1000 women screened among women aged 45 to 69 years, 2006-2022, by DCIS grade.
